# Circular excision *versus* fusiform excision in the treatment of non-melanoma skin cancer: clinical trial on aesthetic and oncological outcomes^؆^

**DOI:** 10.1016/j.abd.2025.501235

**Published:** 2025-11-03

**Authors:** Nathalie Andrade Sousa, Elisa de Oliveira Barcaui, Carla Jorge Machado, Carlos Baptista Barcaui

**Affiliations:** a Department of Dermatology, Universidade do Estado do Rio de Janeiro, Rio de Janeiro, RJ, Brazil; b Department of Dermatology, Universidade Federal do Estado do Rio de Janeiro, Rio de Janeiro, RJ, Brazil; c Department of Preventive and Social Medicine, Universidade Federal de Minas Gerais, Belo Horizonte, MG, Brazil

Dear Editor,

Skin cancer is the most common malignant neoplasm in Brazil and worldwide.^1^, ^2^, ^3^ Several therapeutic modalities are available; however, excision with margins recommended in the literature is the gold standard for treatment.^3^, ^4^, ^5^ Elliptical or fusiform excision is the most commonly used in clinical practice, mainly due to its practicality.^6^, ^7^ However, studies show that this approach can sacrifice a large area of ​​healthy skin by removing three to four times more tissue than the original tumor size, resulting in long and often unsightly scars.^6^, ^8^ In contrast, the literature proposes circular excision as an alternative, with the potential to preserve a larger tissue area, resulting in shorter scars and better alignment with skin tension lines.^6^, ^8^ Given this scenario, the present study aims to compare the fusiform and circular excision techniques in the treatment of low-risk basal cell carcinomas (BCC) and squamous cell carcinomas (SCC), evaluating the length and final scar orientation, with an emphasis on aesthetic and oncological outcomes.

A clinical trial was conducted with 67 patients undergoing surgical excision of lesions suspected of non-melanoma skin cancer, evaluated clinically and dermoscopically by dermatologists with at least five years of training and clinical experience. All patients underwent circular excision, with the material subsequently being sent for conventional histopathological analysis.

Patients ≥ 18 years of age with primary lesions suspected of BCC or SCC, with a diameter ≤ 2 cm, located on the trunk or limbs, were included. Patients < 18 years of age, with recurrent lesions, lesions > 2 cm, located on the head, neck, acral, or genital regions, or without clinical and dermoscopic criteria for BCC or SCC were excluded.

The primary outcome was the size of the scar obtained by circular excision and its final orientation. The secondary outcome was the difference between the planned fusiform excision diameter and the final scar size. The sample size calculation was based on the Hudson-Peacock study,^6^ estimating that at least 63 patients would be needed to detect a 21% difference in scar length with 95% confidence and a 10% margin of error.

The procedure consisted of: 1) Asepsis with 2% chlorhexidine disinfectant and 0.5% alcohol; 2) Circular marking of the lesion with recommended margins; 3) Delimitation of a theoretical zone with a 3:1 length-to-width ratio; 4) Measurement of the zone with a millimeter ruler; 5) Local anesthesia with an anesthetic solution of 20 mL of 2% lidocaine diluted in 20 mL of 0.9% saline and 0.02 mL of epinephrine 1:1000; 6) Excision with a 15-blade scalpel; 7) Hemostasis with electrocautery and tissue dissection; 8) Correction of “dog ears” when necessary; 9) Suturing with simple stitches and Mononylon; 10) Measurement of the final scar with a millimeter ruler and photographic record; 11) Comparison between the length and orientation of the final scar with the recommended fusiform design. The steps are outlined in Figure 1, Figure 2.

**Figure 1:**
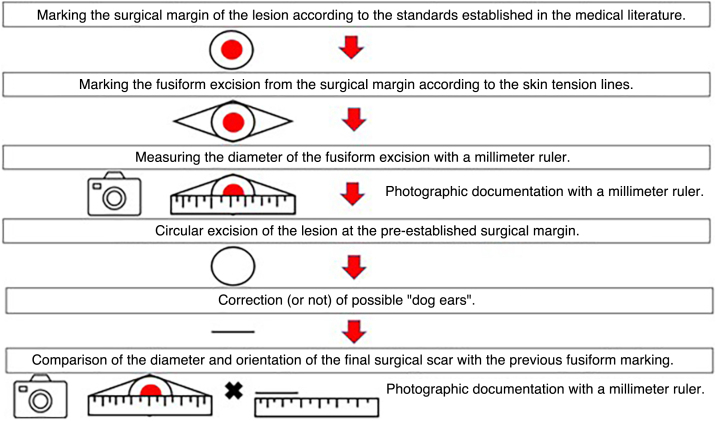
Stages of the surgical procedure.

**Figure 2:**
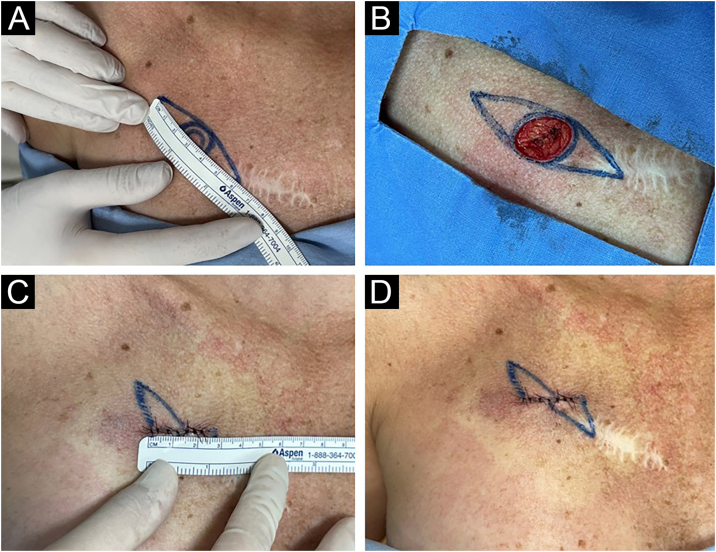
Clinical photographs of the surgical procedure steps. (A) Marking and measuring the fusiform excision diameter from the 0.4 cm circular margin of the lesion according to the skin tension lines. (B) Surgical defect after excision of the lesion at the circular margin. (C) Measurement of the final scar for comparison with the programmed fusiform excision diameter. (D) Final scar: smaller and with a different orientation than the fusiform design.

Clinical, demographic, and surgical data were recorded on a standardized form and transferred to a spreadsheet (Microsoft Excel®). Categorical variables such as sex, age, comorbidities, lesion location, histopathological diagnosis, and final scar orientation were analyzed using the chi-square or Fisher's exact test. Ordinal variables such as age, lesion size, planned fusiform excision diameter, final scar size, and the difference between the fusiform excision size and the final scar size were evaluated using ANOVA or Kruskal-Wallis tests, according to normal or non-normal distribution, respectively. Normality was assessed using the Shapiro-Wilk test.

After histopathological analysis, 63 of the 67 lesions were confirmed as BCC or SCC. Four lesions were excluded due to alternative diagnoses. All removed lesions had neoplasia-free margins. The final sample consisted of 32 women (50.8%) and 31 men (49.2%), with a mean age of 65.3 years. Most lesions were located on the trunk (63.5%). Their diameter ranged from 0.4 to 2.0 cm, with a mean of 1.1 cm. The additional margin used was 0.4 cm for suspected BCC and 0.5 cm for SCC. The mean theoretical zone length was 5.5 cm, while the final scar length was 3.1 cm, representing a mean reduction of 2.4 cm (43.6%). This reduction was observed in 100% of cases. In 32 cases (50.8%), correction of “dog ears” was necessary, 14 unilateral and 18 bilateral, and the remaining 49.2% did not require correction. In 30.2% of surgeries, there was a change in scar orientation, resulting in better alignment with skin tension lines. These data are shown in Table 1.

**Table 1:** Comparative analysis of variables with correction of “dog ears”.

		Number of corrected ears			
	Total	0	1	2	p-value
**Characteristics**		n = 31	n = 14	n = 18	
**Sex, n (%)**					
Female	32 (50.8%)	15 (48.4%)	8 (57.1%)	9 (50.0%)	0.801
Male	31 (49.2%)	16 (51.6%)	6 (42.9%)	9 (50.0%)	
**Age (ordinal)**					
Mean (Standard deviation)	65.3 (14.4)	66.4 (11.7)	66.4 (19.2)	62.7 (14.9)	0.664
Median (Interquartile Interval)	67 (17)	69 (15)	66 (21)	64 (14)	0.676
Minimum; Maximum	29; 92	29; 89	30; 92	29; 85	‒
**Age (categorical), n (%)**					
< 60	18 (28.6%)	6 (19.4%)	5 (35.7%)	7 (38.9%)	0.573
≥ 60	45 (71.4%)	25 (80.6%)	9 (64.3%)	11(61.1%)	
**Histopathological diagnosis, n (%)**					
Basal cell carcinoma	49 (77.8%)	26 (88.9%)	11 (78.6%)	12(66.7%)	0.389
Squamous cell carcinoma	14 (22.2%)	5 (11.1%)	3 (21.4%)	6 (33.3%)	
**Lesion location, n (%)**					
Limbs	23 (36.5%)	10 (32.3%)	4 (28.6%)	9 (50.0%)	0.364
Trunk	40 (63.5%)	21 (67.7%)	10 (71.4%)	9 (50.0%)	
**Lesion size (cm)**					
Mean (Standard deviation)	1.1 (0.4)	1.0 (0.4)	1.2 (0.4)	1.1 (0.5)	0.322
Median (Interquartile Interval)	1.0 (0.6)	0.9 (0.6)	1.2 (0.5)	1.0 (1.0)	0.286
Minimum; Maximum	0.4; 2.0	0.4; 2.0	0.5; 1.8	0.4; 2.0	‒
**Diameter: programmed fusiform excision (cm)**					
Mean (Standard deviation)	5.5 (1.4)	5.2 (1.3)	5.5 (1.5)	5.8 (1.6)	0.350
Median (Interquartile Interval)	5.5 (1.5)	5.0 (2.0)	6.0 (2.0)	6 (2.5)	0.522
Minimum; Maximum	3.0; 9.0	3.0; 8.0	3.0; 8.0	3.4; 9.0	‒
**Final size: scar (cm)**					
Mean (Standard deviation)	3.1 (1.0)	2.5 (0.7)	3.6 (0.9)	3.8 (1.0)	<0.001
Median (Interquartile Interval)	4.0 (1.7)	2.4 (1.0)	3.5 (1.2)	4.0 (0.9)	<0.001
Minimum; Maximum	1.4; 6.0	1.4;4.1	2.3; 5.0	1.5; 6.0	‒
**Programmed fusiform excision diameter – Final scar size (cm)**					
Mean (Standard deviation)	2.4 (1.1)	2.7 (0.9)	2.0 (1.1)	2.0 (1.1)	0.024
Median (Interquartile Interval)	2.5 (1.5)	2.5 (1.5)	2.3 (2.0)	1.8 (2.0)	0.367
Minimum; Maximum	0.2; 5.0	1.1;5.0	0.2;3.7	0.2; 5.0	
**Final scar orientation, n (%)**					
Same	44 (69.8%)	22 (71.0%)	10 (71.4%)	12 (66.7%)	0.938
Different	19 (30.2%)	9 (29.0%)	4 (28.6%)	6 (33.3%)	

The results of this study reinforce that circular excision is an effective alternative to the fusiform technique in the treatment of low-risk BCC and SCC. The reduction in scar length (43.6%) observed in all cases demonstrates that it is possible to preserve healthy tissue without compromising oncological cure, considering that all margins were free of neoplasia.

Compared with the literature, the results were superior to those of the studies by Hudson-Peacock and Lawrence, Seo et al., and Lee et al.^6^, ^8^, ^9^ In these studies, scar reduction ranged from 21% to 35%, with an improvement in the final wound orientation.

The reduced need for “dog ears” correction in about half of the cases is another favorable factor, reducing surgical time and tissue trauma. Furthermore, the possibility of realigning the final scar with skin tension lines in one-third of cases may contribute to a better aesthetic and functional result.

The study has limitations, such as the lack of a control group with true fusiform excision and the exclusion of lesions > 2 cm, which tend to form larger defects, leading to greater tension during closure, which could alter the outcome. Furthermore, the lack of subjective aesthetic evaluation and long-term follow-up are factors to be considered in future studies to validate and expand the findings.

Therefore, circular excision proved to be a safe and effective technique with significant aesthetic advantages in the treatment of low-risk non-melanoma skin cancer. Its application resulted in smaller and, in many cases, better-oriented scars, without compromising oncological treatment, representing a viable alternative to the fusiform technique.

## ORCID ID

Elisa O. Barcaui: 0000-0002-9487-7860

Carla J. Machado: 0000-0002-6871-0709

Carlos B. Barcaui: 0000-0002-3303-3656

## Financial support

None declared.

## Authors' contributions

Nathalie A. Sousa: Study concept; Data curation; Investigation; Project administration; Validation; Visualization; Writing – original draft.

Elisa O. Barcaui: Project administration; Validation; Visualization; Writing – review and editing.

Carla J. Machado: Data curation; Formal analysis; Methodology.

Carlos B. Barcaui: Study concept; Data curation; Supervision; Project administration; Validation; Visualization; Writing – review and editing.

## Availability of research data

The entire dataset supporting the results of this study was published in the article itself.

## Conflicts of interest

None declared.
